# Supplementary material for: An Improved Protocol for Agrobacterium-Mediated Transformation in Subterranean Clover (Trifolium subterraneum L.)
Source: Int J Mol Sci. 2021 Apr 17;22(8):4181. doi: 10.3390/ijms22084181 (PMC8073064; doi:10.3390/ijms22084181)
Supplement: Supplementary file 1 [file ijms-22-04181-s001.zip › ijms-1172838-supplementary.pdf]

Table S1. Summary of reagents used.

Description	Provider	Order in Text
Tween-20	Amresco	1
Cefotaxime	PhytoTechnology Laboratories	2
Thidiazuron (TDZ)	PhytoTechnology Laboratories	3
Naphthaleneacetic acid (NAA)	PhytoTechnology Laboratories	4
1.2 μM indole-3-butyric acid (IBA)	PhytoTechnology Laboratories	5
Indole-3-acetic acid (IAA)	Sigma-Aldrich	6
Kinetin	Sigma-Aldrich	7
Agar	PhytoTechnology Laboratories	8
Kanamycin	PhytoTechnology Laboratories	9
Luria Bertani medium	Sigma-Aldrich	10
Streptomycin	Sigma-Aldrich	11
Spectinomycin	Sigma-Aldrich	12
Acetosyringone	Sigma-Aldrich	13
Phire® Plant Direct PCR Kit	Thermo Scientific	14
